# Whole Peptidoglycan Extracts from the* Lactobacillus paracasei* subsp.* paracasei* M5 Strain Exert Anticancer Activity* In Vitro*

**DOI:** 10.1155/2018/2871710

**Published:** 2018-02-07

**Authors:** Shumei Wang, Xue Han, Lanwei Zhang, Yingchun Zhang, Hongbo Li, Yuehua Jiao

**Affiliations:** ^1^Key Laboratory of Dairy Science of Ministry of Education, Northeast Agricultural University, Harbin 150030, China; ^2^School of Chemical Engineering & Technology, Harbin Institute of Technology, Harbin 150090, China; ^3^School of Food Science and Engineering, Ocean University of China, Qingdao 266100, China

## Abstract

The* Lactobacillus paracasei *subsp*. paracasei *M5 strain exerted potential anticancer activity through the cell wall. In this study, whole peptidoglycan (WPG) was extracted from the* Lactobacillus paracasei *subsp*. paracasei* M5 strain and was evaluated for anticancer effects as well as its properties. SDS-PAGE analysis confirmed the presence of WPG with dominant bands of approximately 14.4 kDa. Further analysis revealed that the amino acids present in the WPG consisted of alanine, glycine, glutamic acid, and lysine in a molar ratio of approximately 8 : 5 : 3 : 3.5. In addition, the cell viability of HT-29 cells with WPG addition was investigated with methyl thiazolyl tetrazolium (MTT) and trypan blue exclusion (TBE) assays, and cell apoptosis was analyzed with a transmission electron microscope, flow cytometry, and semiquantitative RT-PCR. These results showed that WPG exerted cytotoxic effects on colon cancer HT-29 cells in a dose-dependent manner and upregulated proapoptotic genes, while downregulating antiapoptotic genes. The gene expression study definitively revealed that WPG induced a mitochondria-mediated apoptotic pathway.

## 1. Introduction

Colon cancer is one of the most common types of cancer with a high incidence in developed countries [1]. Previous studies have shown that there are several hundred different bacterial species present in the human colon and some colonic microbiota have been implicated to promote human health, while the other members of the colonic microbiota have been shown to induce colorectal cancer [2]. Probiotic, a live microbial ingredient that is beneficial to health, plays an important role in inhibiting tumors [3, 4]. Among probiotics,* Lactobacillus* species are probably the best studied microorganisms at present [5]. The metabolites of* lactobacilli* can probably exert a crucial role in probiotic function among their special mechanisms [6]. For this reason, the ability of several natural products, whole cells, heat-killed cells, cell wall, and cytoplasm fractions from lactobacilli to prevent tumor have been studied in detail [6–10]. The intact cell wall structure of* lactobacilli*, for instance, could activate innate immunity and remarkably inhibit the growth of various cancer cells [6, 7, 11]. Our group previously suggested that the heat-killed cells and cell wall extracted from the M5 strain exerted significant antiproliferative activity against HT-29 cells. The Gram-positive cell wall mainly consists of peptidoglycan (approximately 90%) [12, 13]. Therefore, we presumed that the anticancer activity of the cell wall from* Lactobacillus paracasei *subsp*. paracasei* M5 strain might be attributed to peptidoglycan fractions. Recently, apoptosis-inducing compounds isolated from bacterial strains have been reported in many studies [14, 15]. Therefore, this study was performed to investigate our hypothesis by detecting the whole peptidoglycan- (WPG-) induced apoptosis in colon cancer HT-29 cells. To the best of our knowledge, there is no previous information about the anticancer activity of WPG, which was isolated from whole cells and retained the intact cell wall structure. The objectives of this paper were to elucidate the* Lactobacillus paracasei* subsp*. paracasei *M5 strain anticancer mechanism with WPG. In addition, we confirmed the elemental property of WPG of the* Lactobacillus paracasei *subsp*. paracasei* M5 strain.

## 2. Materials and Methods

### 2.1. Lactobacillus Strain and Culture Conditions

The* Lactobacillus paracasei *subsp*. paracasei* M5 strain was obtained from traditional koumiss in Sinkiang, China [10], and was selected in this study on the basis of its high adherence to human colonic epithelial cells [16], immunomodulatory activity [17], and antiproliferative activity against human colon cancer cell line HT-29 [10, 12]. The* Lactobacillus paracasei *subsp*. paracasei* M5 strain was cultured in de Man, Rogosa, and Sharpe (MRS) (Difco) broth (Aladdin, China) with 0.05% (w/w) L-cysteine at 37°C under anaerobic conditions. The strain was subcultured twice at 37°C for 18 h before use.

### 2.2. Preparation of WPG from Lactobacillus Strain

WPG was extracted from the* Lactobacillus paracasei* subsp.* paracasei* M5 strain according to the method in Sekine et al. [18]. The protein content of WPG was determined by the coomassie brilliant blue method and WPG was stored at −80°C until use.

### 2.3. SDS-PAGE Analysis of WPG

The WPG was suspended in 100 mL of loading buffer and boiled for 5 min. Gel electrophoresis with sodium dodecyl sulfate on 10% polyacrylamide was performed to compare the WPG. Bio-Rad SDS-PAGE broad-range molecular markers with molecular masses ranging from 14.4 to 116.0 kDa were used.

### 2.4. Amino Acid Composition Analysis of WPG

WPG (1.5 mL) at a concentration of 1 mg mL^−1^ was mixed with 1.5 mL of 6 M HCl. After sealing the ampoule, the WPG was hydrolyzed under nitrogen at 110°C for 24 h. The resulting solution was mixed with 1.5 mL of 6 M NaOH for neutralization and then adjusted to 5 mL with 0.02 M HCl. Amino acid compositions were measured using a Hitachi L-8800 amino acid analyzer (Hitachi Corp., Japan) [16].

### 2.5. Morphologic Observation of WPG

Morphologic observation of WPG was performed in Northeast Agricultural University of Life Science Center. Briefly, WPG was immersed in 2% glutaraldehyde at 4°C and was then prepared for investigation using scanning electron microscopy (S-3400N, Hitachi Corp., Japan) [19]. In addition, WPG was fixed with 2% glutaraldehyde for 2 h at room temperature (25°C) and was dehydrated by successive treatment with methanol [18]. Electron micrographs were collected with a Hitachi H-7650 transmission electron microscope (Hitachi Corp., Japan).

### 2.6. Cell Cultures

A human colon cancer HT-29 cell line was obtained from the Cancer Institute of the Chinese Academy of Medical Science (Beijing, China). HT-29 cells were routinely cultured in a 75-cm^2^ flask containing complete RPMI-1640 medium (Hyclone, Utah, USA) supplemented with 10% (v/v) fetal bovine serum (Sijiqing Co. Ltd., Zhejiang, China) and 1% (v/v) penicillin-streptomycin antibiotics (10,000 IU mL^−1^ and 10,000 *μ*g mL^−1^; Gibco, New York, USA) with 5% CO_2_ and 95% air at 37°C.

African green monkey kidney cells (Vero cells), which were defined as a continuous cell line with a fibroblastic-like morphology, were obtained from Harbin Veterinary Research Institute (Harbin, China). Vero cells were routinely cultured in Dulbecco's Modified Eagle medium supplemented with 10% (v/v) heat inactivated fetal calf serum and 1% (v/v) antibiotics penicillin-streptomycin (10,000 IU mL^−1^ and 10,000 *μ*g mL^−1^) with 5% CO_2_ and 95% air at 37°C.

### 2.7. Measurement of Antiproliferative Activity

The antiproliferative activity was tested via the methyl thiazolyl tetrazolium (MTT) and trypan blue exclusion (TBE) assays. HT-29 and Vero cells (1 × 10^5^ cells) were seeded in 96-well microtiter plates (Corning Inc., Corning, NY) at 100 *μ*L per well and incubated at 37°C for 24 h. Test samples of WPG from the* Lactobacillus paracasei *subsp*. paracasei* M5 strain at various concentrations (10 *μ*g mL^−1^, 20 *μ*g mL^−1^, 40 *μ*g mL^−1^, 80 *μ*g mL^−1^, and 160 *μ*g mL^−1^) were added to the wells. Positive (5-Fu) and negative (purified water) controls were included for all experiments. The MTT assay was examined as described by Tuo et al. [10]. The TBE assay was examined as described by Thirabunyanon et al. [9]. Each assay was repeated in triplicate. The results were presented as the inhibition rates; all results were transformed into percentages based on their respective controls. Values were calculated using the following equations:

(1) Inhibition  rate=1−absorbance  in  test  wellabsorbance  in  control  well×100%  MTT,Inhibition  rate=dead  cell  counttotal  cell  count×100%  TBE.

### 2.8. Morphologic Observation of Apoptosis

HT-29 cells (1 × 10^6^ cells) were seeded in 6-well plates (Corning Inc., Corning, NY) with 80 and 160 *μ*g mL^−1^ WPG at 37°C for 48 h. After incubation, HT-29 cells were fixed with 2.5% glutaraldehyde for 24 h at 4°C, which was followed by fixation with 1% osmium tetroxide fixation for 1 h at room temperature (25°C). Then, HT-29 cells were dehydrated by acetone series (30%, 50%, 70%, 90%, and 100%). Ultrathin sections were stained with uranyl acetate and lead citrate and were then observed under a Hitachi H-7650 transmission electron microscope (Hitachi Corp., Japan).

### 2.9. Cell Cycle Distribution by Flow Cytometry

HT-29 cells (1 × 10^6^ cells) were seeded in 6-well plates with 80 and 160 *μ*g mL^−1^ WPG. After 48 h, the cells were collected and fixed with 1 mL of ice-cold 70% ethanol at 4°C overnight. After fixation, cells were washed twice with ice-cold phosphate buffer saline (PBS, pH 7.2) and were then stained with 200 *μ*L propidium iodide (PI) and RNase A in the dark for 30 min. The cell cycle distribution was then analyzed by flow cytometry (FACSCalibur, USA) [20]. The proportion of nuclei at each phase of the cell cycle was obtained using ModFit LT TM DNA analysis software.

### 2.10. Measurement of Apoptosis

HT-29 cells (5 × 10^5^ cells) were seeded in 6-well plates with 80 and 160 *μ*g mL^−1^ WPG. After incubation for 48 h, the cells were washed with ice-cold PBS (pH 7.2) and stained with 5 *μ*L of Annexin V-FITC and 5 *μ*L of PI for 15 min at room temperature (25°C) in the dark. HT-29 cells were analyzed within one hour by flow cytometry (FACSCalibur, USA) equipped with a 488 nm argon laser light source, 515 nm band pass filter for FITC fluorescence, and 633 nm band pass filter for PI fluorescence. The experimental data were further analyzed by Cell Quest software [21].

### 2.11. Measurement of the Mitochondrial Membrane Potential

HT-29 cells (5 × 10^5^ cells) were seeded in 6-well plates with 80 and 160 *μ*g mL^−1^ WPG. After 48 h of incubation, HT-29 cells were washed with ice-cold PBS (pH 7.2) and stained with 15 *μ*L of rhodamine 123 (R123) for 30 min at room temperature (25°C) in the dark [22]. The cells were measured by flow cytometry (FACSCalibur, USA) and data were analyzed using Cell Quest software.

### 2.12. Total RNA Isolated and RT-PCR Analysis

The expressions of apoptosis related genes, Bcl-xl, Bax, Bad, caspase-3, and Cytochrome c (Cyto-C), in HT-29 cells were studied using semiquantitative RT-PCR. The *β*-actin housekeeping gene was used as control. Total RNA was isolated from the cells using a total RNA isolation kit (BioFlux, BSC52M1). cDNA was generated from 2 *μ*g of total RNA using a reverse transcription kit (AE401, Transgen Biotech, China) in accordance with the manufacturer's instructions. The forward and reverse primers of apoptotic genes are shown in Table 3. The PCR conditions were 94°C for 2 min, which was followed by 35 cycles of 94°C for 15 s, 55°C for 30 s, annealing at 68°C for 60 s, and a final extension at 72°C for 5 min. Finally, PCR products were separated by 1.5% agarose gel and stained with ethidium bromide. Gene expression was quantified by densitometry using image analysis software (Quantity One; Bio-Rad, Hercules, CA, USA). The *β*-actin gene was used as an internal control and its expression was considered 100%.

### 2.13. Cytochrome c Assay

HT-29 cells (5 × 10^5^ cells) were seeded in 6-well plates and treated with different concentrations of WPG. After 48 h, cells were centrifuged (500 ×g, 10 min) and washed twice with ice-cold PBS (pH 7.2). Cell proteins were extracted on ice using cell extraction buffer (P0013B, Beyotime, Shanghai, China). Cyto-C in the cell proteins was analyzed using an Enzyme Linked-Immuno-Sorbent Assay kit (KHO1051, Invitrogen, Carlsbad, CA, USA) according to the manufacturer's instructions.

### 2.14. Statistical Analysis

All experiments were performed in triplicate. The results were expressed as the means ± standard deviation. Statistical analysis was performed with SPSS14.0 for Windows (SPSS Inc., Chicago, IL). One-way ANOVAs with Duncan's post hoc test were used. A probability level of *P* < 0.05 was used throughout this study.

## 3. Results and Discussion

### 3.1. The Elemental Property of WPG

**Figure 1:**
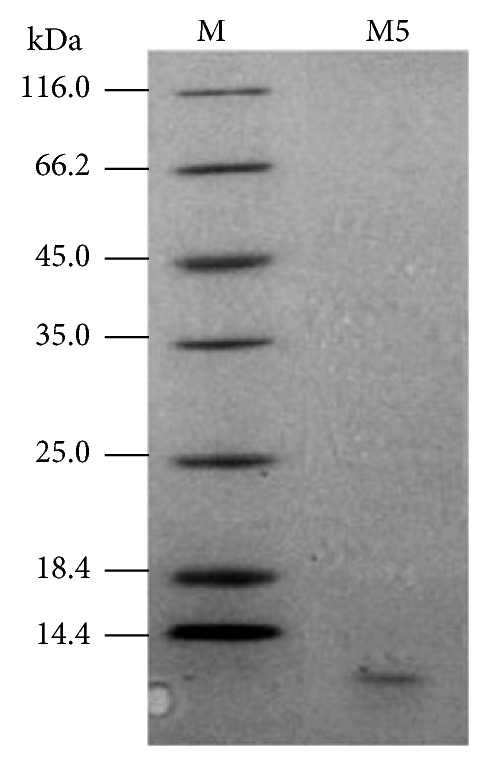
SDS-PAGE analysis of WPG proteins extracted from* Lactobacillus paracasei *subsp*. paracasei* M5 strain.

SDS-PAGE analysis revealed the presence of WPG, and the major molecular masses were approximately 14.4 kDa (Figure 1). Sekine et al. [18] claimed that WPG had a unique, physically intact skeleton structure for the cell wall of most bacteria [23], which consisted of chains of peptidoglycan monomers that were cross-linked by short peptide bridge. The monomers consisted of alternating N-acetylglucosamine (G) and N-acetylmuramic acid (M) residues [23]. The composition of the peptide bridge consists of several amino acid residues, such as alanine, glutamic acid, glycine, and lysine. To obtain detailed information, we analyzed the amino acid compositions of WPG. The approximate molar ratio of alanine, glutamic acid, glycine, and lysine was 8 : 5 : 3 : 3.5 (Table 1). Similarly, peptidoglycan contained alanine, glutamic acid, and lysine in a molar ratio of approximately 1.2 : 1 : 1 [24]. Generally speaking, the molar ratio of the amino acids is different in peptidoglycans from different strains [24]. However, alanine, glutamic acid, glycine, and lysine, which are common primary ingredients in peptidoglycan, were found in cell wall preparations from* Micromonospora* species [25] and in peptidoglycan fractions from the* Methanobacterium* genus [24].

**Figure 2:**
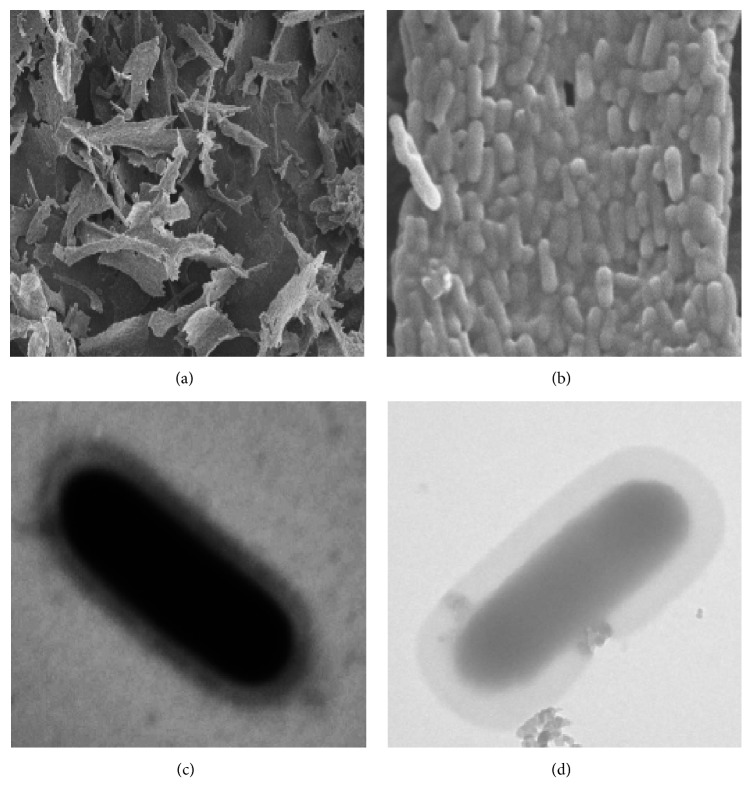
WPG extracted from* Lactobacillus paracasei *subsp*. paracasei* M5 strain was investigated via scanning electron microscope, original magnification 1000x (a) and original magnification 11000x (b). The whole cell (c) and WPG (d) from M5 strain were investigated via transmission electron microscope, original magnification 10000x.

Sekine et al. suggested that WPG, which was isolated from whole cells without being subjected to physically destructive methods, completely retained the intact cell wall structure [18]. In our study, scanning electron microscopy (Figures 2(a) and 2(b)) and transmission electron microscopy (Figure 2(d)) revealed that the physical structure of WPG was invariant during the isolation and chemical purification procedures and completely retained the shape of the whole cells (Figure 2(c)). Similarly, WPG of* Micromonospora* [25] and genus* Methanobacterium* [24] were morphologically indistinguishable from whole cells, which had the skeletal structure integrity of* lactobacilli* cell wall [18].

### 3.2. Effects of WPG on the Growth of HT-29 and Vero Cells

**Figure 3:**
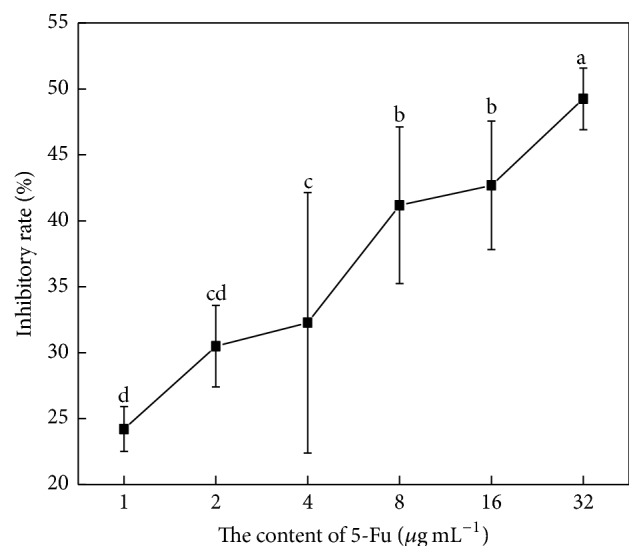
Inhibitory rate (%) of 5-Fu (positive control) exerted on colon cancer HT-29 cells. Values with different letters are significantly different (*P* < 0.05).

**Figure 4:**
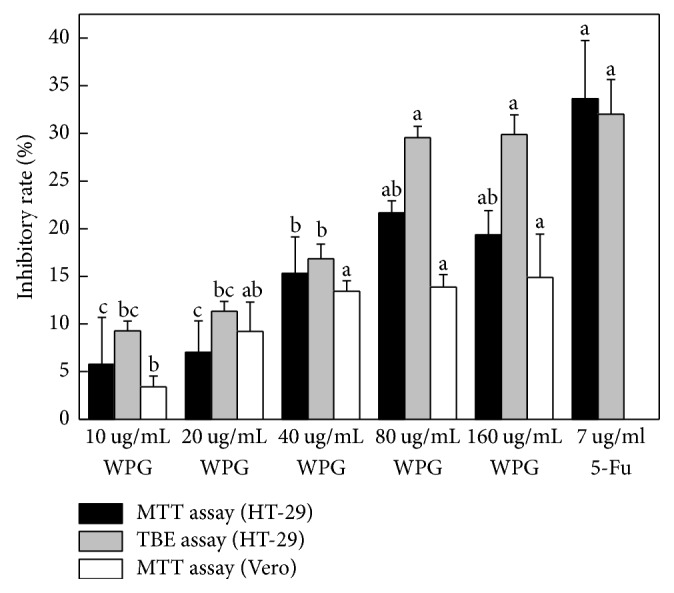
Comparison of antiproliferative effects caused by WPG on human colon cancer HT-29 cells and Vero cells. Cells were exposed to different concentrations of WPG for 48 h and inhibitory effects were determined by MTT assay and TBE assay. Positive control was 5-Fu and negative control was purified water. Values with different letters on the columns are significantly different (*P* < 0.05).

In the present study, we tested the antiproliferative activity of WPG from the* Lactobacillus paracasei *subsp*. paracasei* M5 strain via MTT and TBE assays. 5-Fu (10.31 *μ*g mL^−1^) had concentrations that produced approximately 50% inhibitory effects on the HT-29 cells (IC_50_) via the MTT assay, which was the toxicity dose in our tests (as shown in Figure 3). Therefore, 7 *μ*g mL^−1^ (lower than 10.31 *μ*g mL^−1^) of 5-Fu was used as a positive control in these experiments [26]. Exposure of HT-29 cells to increasing concentrations of WPG showed significant antiproliferative activity after 48 h of treatment.  Figure 4 showed that increasing concentrations of WPG caused a significant reduction in the conversion of the MTT tetrazolium salt by live HT-29 cells, but it was not significantly higher than 5-Fu (*P* < 0.05). Similarly, 100 *μ*g mL^−1^ cytoplasmic fraction from* Lactobacillus* inhibited the proliferation of SNU-1 stomach adenocarcinoma cells [27].

To further confirm the antiproliferative activity, WPG was monitored using the TBE assay. There was no significant difference between the inhibitory rates from 80 *μ*g mL^−1^, 160 *μ*g mL^−1^ of WPG, and 7 *μ*g mL^−1^ of 5-Fu via the TBE assay (Figure 4). The results of antiproliferative activity from the TBE assay agreed with the MTT assay. On the whole, the antiproliferative activity of WPG was dose dependent and the strongest antiproliferative activity was observed at 80 *μ*g mL^−1^ and 160 *μ*g mL^−1^ of WPG. The results agreed with those reported from Kim et al. [5] that peptidoglycans from lactic acid bacteria at a concentration of 100 *μ*g mL^−1^ had significant antiproliferative activity and from Fichera and Gunter [28] that peptidoglycan from* L. casei* at different doses decreased the viability of various tumor cell lines by 25–30%.

Many cancer therapy medicines were limited in use because of their toxic effects on noncancerous cells [29]. However, WPG extracted from the* Lactobacillus paracasei *subsp*. paracasei* M5 strain, which was obtained from traditional koumiss, might be safely used as natural cancer therapeutic agents. Haza et al. showed that lactic acid bacteria strains isolated from milk cheese could not affect the viability of Vero cells [30]. Fichera and Gunter also suggested that* L. casei* and its derivative peptidoglycan both have stimulatory activity in normal cells and inhibitory activity in tumor cells [28]. For this reason, we investigated the cytotoxic effects of WPG on noncancerous Vero cells. Interestingly, the antiproliferative effects of WPG on noncancerous Vero cells (inhibitory rate from 3.39% to 14.89% in Figure 4) were significantly lower than that of HT-29 cells (inhibitory rate from 5.76% to 21.65% via the MTT assay and inhibitory rate from 9.27% to 29.89% via the TBE assay in Figure 4). Therefore, only WPG had minor toxic activity on Vero cells when compared with that observed in HT-29 cells. Similar results were observed on other cancer cell lines in the present study [31]. Therefore, WPG extracted from the* Lactobacillus paracasei *subsp*. paracasei* M5 strains may be safely used as a cancer treatment agent because it only exerted minor toxic activity on noncancerous (Vero) cells.

### 3.3. Effects of WPG on HT-29 Cell Morphology

**Figure 5:**
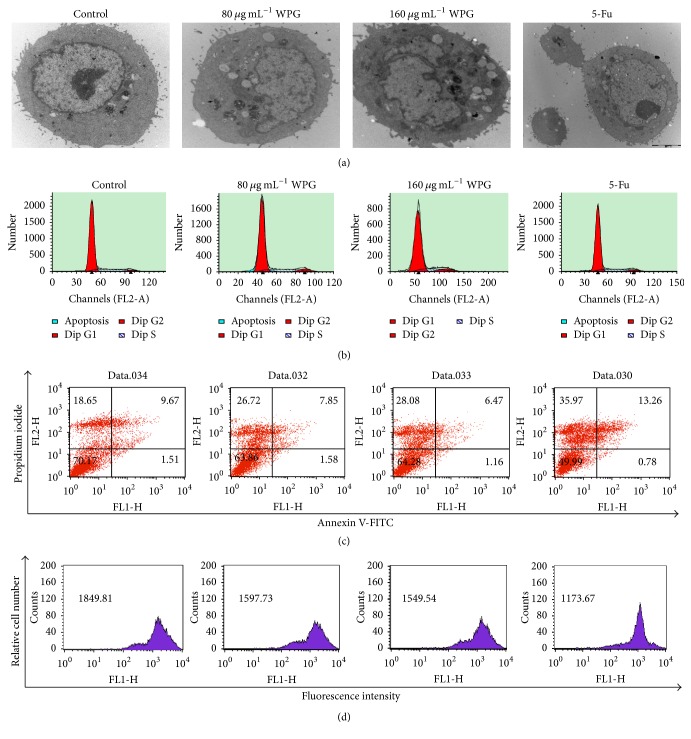
HT-29 cells were treated with 80 and 160 *μ*g mL^−1^ of WPG for 48 h, and the apoptotic cells were investigated via transmission electron microscopic, original magnification 10000x (a). HT-29 cells were stained with PI (b), Annexin V-FITC/PI (c), and Rhodamine 123 (d) and then submitted to flow cytometric analysis. Positive control was 5-Fu and negative control was purified water. HT-29 cells that stained positive for Annexin V-FITC and negative for PI were undergoing apoptosis. The cells that stained positive for both Annexin V-FITC and PI were either in the end stage of apoptosis or already dead. The cells that stained negative for both Annexin V-FITC and PI were viable and not undergoing apoptosis (a).

Apoptosis is a normal physiologic process that is essential for the development and maintenance of tissue homeostasis. Apoptosis is characterized by a series of morphological changes. After treatment with 80 *μ*g mL^−1^ and 160 *μ*g mL^−1^ of WPG for 48 h, HT-29 cells began to show typical morphologic changes (Figure 5(a)). These ultrastructural changes were characteristic of apoptosis, including chromatin condensation, nuclear fragmentation, and apoptotic body formation, which appeared in apoptotic cells. The control cells showed a normal morphology with randomly distributed organelles and a single large electron dense nucleolus with uniformly dispersed chromatin. Chromatin condensation and vacuoles in the cytoplasm were observed in the cells treated with 80 *μ*g mL^−1^ and 160 *μ*g mL^−1^ of WPG. Simultaneously, the chromatin condensation, pseudopods, and morphologic changes were also observed in the cells treated with 7 *μ*g mL^−1^ of 5-Fu. Similarly, the characteristic morphology of HT-29 cells was changed within 96 h after treatment with a soluble polysaccharide fraction extracted from* L. acidophilus* 606 [31]. DNA fragmentation and chromatin condensation of SNU-1 stomach adenocarcinoma cells were found after treatment with 100 *μ*g mL^−1^ cytoplasmic fraction from* Lactobacillus* [27]. There were morphological alterations in human bladder cells after treatment with* L. casei* and its derivative peptidoglycan in vitro [28]. Generally, induction of apoptosis has been recognized as an important anticancer approach. In this study, morphology demonstrated that 80 and 160 *μ*g mL^−1^ of WPG extracted from the* Lactobacillus paracasei *subsp*. paracasei* M5 strain induced apoptosis in HT-29 cells.

### 3.4. WPG Induces Cell Cycle Arrest in HT-29 Cells

To further confirm the effects of WPG on HT-29 cell proliferation and apoptosis, we conducted a cell cycle distribution experiment. Cell growth is regulated through several different stages (G1, S, G2, and M), which ensure its concordant progression. The presence of WPG significantly affected the cell cycle distribution.  Figure 5(b) and Table 2 showed when HT-29 cells were treated with 80 and 160 *μ*g mL^−1^ of WPG and 7 *μ*g mL^−1^ of 5-Fu for 48 h. The percentages of cells increased 2.65, 3.09, and 1.21% in G2 phase and decreased 0.55, 2.84, and 1% in S phase compared with the untreated control cells (*P* < 0.05). However, no change was observed in the G1 phase. These observations indicated that WPG from the* Lactobacillus paracasei *subsp*. paracasei* M5 strain induced G2 phase arrest, which might be involved in the inhibition of HT-29 cell proliferation. Similarly, it has also been reported that a 100 *μ*g mL^−1^ cytoplasmic fraction of* L. lactis *subsp*. lactis* inhibited stomach cancer SNU-1 cell growth through inducing G0/G1 cell cycle arrest [27] and inhibited colon cancer SNUC2A cells growth through inducing S phase accumulation [32].

### 3.5. WPG Induces Cells Apoptosis in HT-29 Cells

In apoptotic cells, the membrane phosphatidylserine (PS) is translocated from the inner side of the plasma membrane to the cell surface. This process is marked by Annexin V binding to cells with exposed PS and is followed by permeabilized membrane and later compromised membrane marked by PI intercalation [21]. Annexin V-FITC staining precedes the loss of membrane integrity that accompanies the later stages of cell death, resulting from either apoptotic or necrotic processes. In this study, for the control cells, 70.17% of the cell population was viable, 1.51% of cells were in early apoptosis, and 18.65% of cells were in necrosis (Figure 5(c)). In cells treated with 80 and 160 *μ*g mL^−1^ of WPG for 48 h, there was 8.07–9.43% increase in necrosis (*P* < 0.05), but there was no increase in early apoptosis compared with untreated cells. Seow et al. showed* Lactobacillus casei *Shirota induced bladder cancer cell death primarily via necrosis, and no apoptotic cells were detected after treatment with* L. casei *Shirota [33]. These results suggested that WPG stimulated apoptosis in the colon cancer HT-29 cells.

### 3.6. WPG Induces ΔΨ_*m*_ Loss in HT-29 Cells

Mitochondria play an important role in maintaining cellular integrity and function [21]. Inducing ΔΨ_*m*_ loss is generally known to lead to functional alterations and initiation of the apoptotic mitochondrial pathway. To investigate whether mitochondria involved in WPG induced apoptosis, we examined the change of ΔΨ_*m*_ of HT-29 cells treated with WPG.  Figure 5(d) shows 80 and 160 *μ*g mL^−1^ WPG caused breakdown of ΔΨ_*m*_ (252.08 and 300.27) in HT-29 cells compared with untreated control cells (*P* < 0.05). Dissipation of ΔΨ_*m*_ might initiate the mitochondrial apoptotic signaling pathway. WPG, which is an exoskeleton and surface that interacts with the environment, plays an essential role in the expression of tumor-regressive activity [18, 24]. In the present study, WPG extracted from the* L. paracasei *subsp*. paracasei* M5 strain exerted strong anticancer activity against the proliferation of HT-29 cells, which is evidenced by apoptosis and breakdown of ΔΨ_*m*_ in HT-29 cells.

### 3.7. Expression of Apoptotic Genes and Cyto-C Release of HT-29 Cells

**Figure 6:**
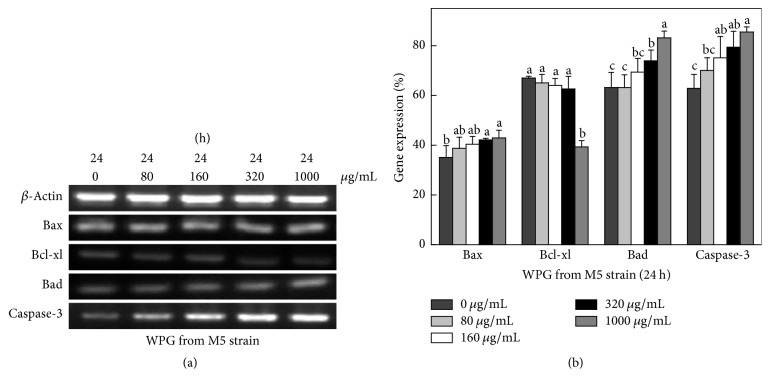
Expression of pro- and antiapoptotic genes in HT-29 cells following exposure to WPG for 24 h. (a) Semiquantitative RT-PCR analysis of apoptotic signaling genes. Column 1, control cells treated with purified water; Column 2, 80 *μ*g mL^−1^ of WPG treated cells; Column 3, 160 *μ*g mL^−1^; Column 4, 320 *μ*g mL^−1^; Column 5, 1000 *μ*g mL^−1^. Results were confirmed by several separate experiments and representative images are shown. (b) Quantitative expression of apoptotic signaling genes (Bax, Bcl-xl, Bad, and caspase-3) compared with the housekeeping gene *β*-actin (expression of *β*-actin was considered 100%). Values with different letters on the columns are significantly different (*P* < 0.05).

**Figure 7:**
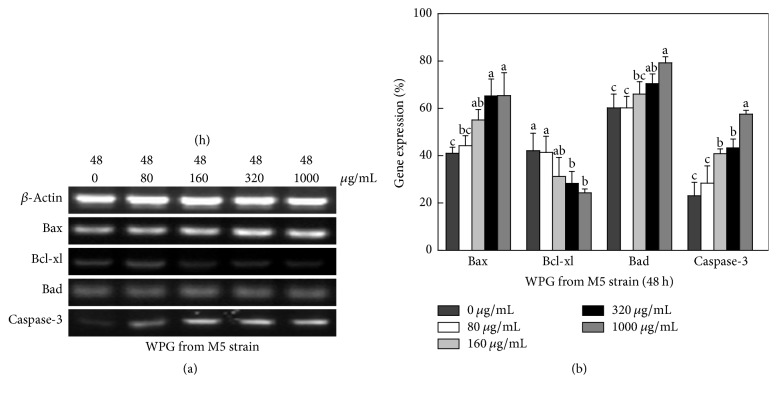
Expression of pro- and antiapoptotic genes in HT-29 cells following exposure to WPG for 48 h. (a) Semiquantitative RT-PCR analysis of apoptotic signaling genes. Column 1, control cells treated with purified water; Column 2, 80 *μ*g mL^−1^ of WPG treated cells; Column 3, 160 *μ*g mL^−1^; Column 4, 320 *μ*g mL^−1^; Column 5, 1000 *μ*g mL^−1^. Results were confirmed by several separate experiments and representative images are shown. (b) Quantitative expression of apoptotic signaling genes (Bax, Bcl-xl, Bad, and caspase-3) compared with the housekeeping gene *β*-actin (expression of *β*-actin was considered 100%). Values with different letters on the columns are significantly different (*P* < 0.05).

**Figure 8:**
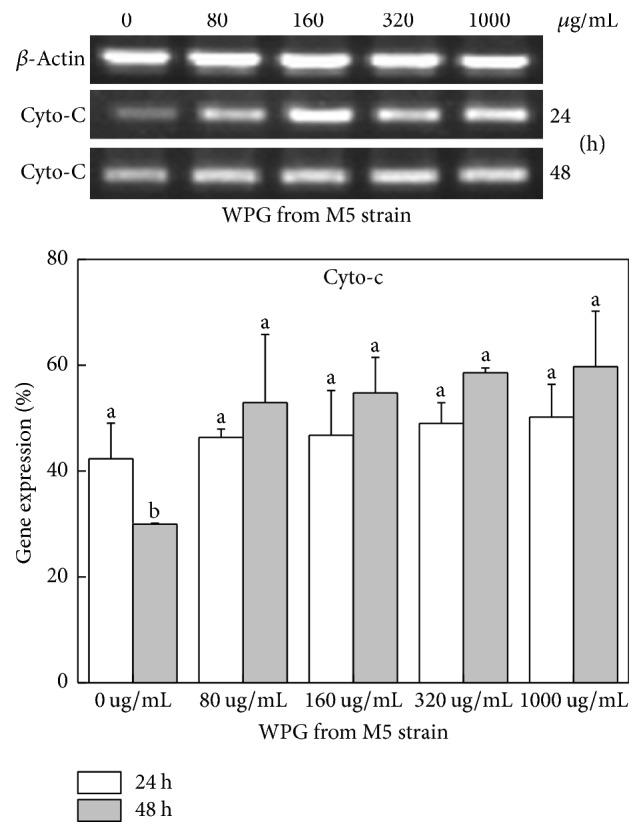
Expression of Cyto-C gene in HT-29 cells following exposure to WPG for 24 or 48 h. Semiquantitative RT-PCR analysis of Cyto-C gene. Column 1, control cells treated with purified water; Column 2, 80 *μ*g mL^−1^ of WPG treated cells; Column 3, 160 *μ*g mL^−1^; Column 4, 320 *μ*g mL^−1^; Column 5, 1000 *μ*g mL^−1^. Results were confirmed by several separate experiments and representative images are shown. Quantitative expression of Cyto-C gene compared with the housekeeping gene *β*-actin (expression of *β*-actin was considered 100%). Values with different letters on the columns are significantly different (*P* < 0.05).

Expression of genes related to apoptosis, including Bcl-xl, Bax, Bad, caspase-3, and Cyto-C, in HT-29 cells was studied using semiquantitative RT-PCR. The housekeeping gene *β*-actin was used as a control. The forward and reverse primers of apoptotic genes are shown in Table 3. Caspases were shown to be activated during apoptosis in many cancer cell lines and played critical roles in initiating apoptosis. This study suggested that the mechanism of WPG induced apoptosis of HT-29 cells involves caspase-3 activation (Figures 6 and 7). Yu et al. reported that caspase-3 was essential for the morphological changes associated with apoptosis [34]. In our experiments, we confirmed the typical morphologic changes and caspase-3 activation in HT-29 cells during apoptosis for cells treated with WPG from the* Lactobacillus paracasei *subsp*. paracasei* M5 strain; the results agreed with the reports from Yu et al. [34]. Cyto-C was shown to be released from the mitochondria into the cytosol during apoptosis in intact cells and was identified as a component required for the crucial steps in apoptosis and caspase-3 activation [35]. This study showed an increase in the Cyto-C gene expression in the cytosol (Figure 8) after treatment with 80–1000 *μ*g mL^−1^ of WPG, and this process occurred via activation of caspase-3 (Figures 6 and 7).

**Figure 9:**
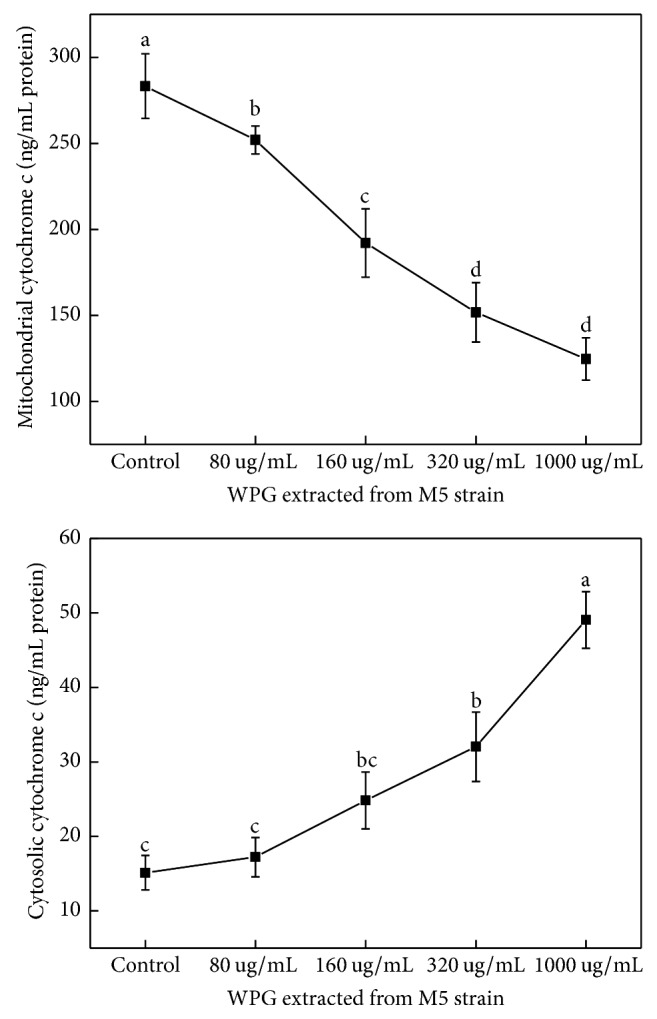
The effect of WPG on the level of mitochondrial and cytosolic Cyto-C. Cyto-C was quantified using an ELISA. Values with different letter are significantly different (*P* < 0.05).

Proteins of the Bcl-2 family were the important regulators of apoptosis [36]. Bcl-xl is an antiapoptotic gene and Bax and Bad are proapoptotic genes in Bcl-2 family. The effects of WPG from* Lactobacillus* on the Bcl-2 family genes appear to be less pronounced. In this study, WPG extracted from the* Lactobacillus paracasei* subsp.* paracasei* M5 strain had increased expression of Bax and Bad genes and decreased expression of the Bcl-xl gene in HT-29 cells after treatment for 24 h (Figure 6) and 48 h (Figure 7). These results confirmed the role of Bax and Bad genes in inhibiting the antiapoptotic function of Bcl-2 and Bcl-xl [37]. Bcl-xl is reportedly downregulated by Cyto-C [37]. This study showed a decreased level of Cyto-C in mitochondria, an increased level of Cyto-C in the cytosol (Figure 9), and a decrease in Bcl-xl gene expression, which confirmed the role of Cyto-C in the downregulation of the Bcl-xl gene. These results clearly indicated that WPG induced apoptosis involved complex interplay of signaling molecules in Cyto-C and the caspase-3 dependent pathway. Accordingly, understanding of gene regulation in apoptotic processes would primarily establish WPG from the* Lactobacillus paracasei* subsp.* paracasei* M5 strain as a potent therapeutic agent for medical applications.

## 4. Conclusion

This study showed that WPG from the* Lactobacillus paracasei* subsp.* paracasei* M5 strain exerted anticancer effects on a human colon cancer HT-29 cell line via antiproliferation and by inducing apoptosis. Simultaneously, WPG exerted only minor toxic activity on a noncancerous Vero cell line. In addition, WPG could upregulate proapoptotic genes and downregulate antiapoptotic genes as well as promote Cyto-C release from the mitochondria into the cytosol. The* Lactobacillus paracasei* subsp.* paracasei* M5 strain, which was a viable candidate, was investigated in our laboratory. We anticipated that the* Lactobacillus paracasei* subsp.* paracasei* M5 strain will have potential applications in functional products in the future.

## Figures and Tables

**Table 1:** The amino acid composition of WPG proteins.

Amino acid	M5 (Molar ratio%)
Aspartic acid	11.35
Threonine	6.23
Serine	4.23
Glutamic acid	10.41
Glycine	6.36
Alanine	15.92
Valine	4.02
Methionine	5.36
Isoleucine	4.30
Leucine	6.62
Tyrosine	2.58
Phenylalanine	2.25
Lysine	7.19
Histidine	2.05
Arginine	3.16

*Note. *The values reported represent the average of two determinations.

**Table 2:** The effect of WPG on the cell cycle distribution.

	G1 (%)	G2 (%)	S (%)
Control	81.20 ± 1.10^a^	2.91 ± 0.03^b^	15.89 ± 0.10^a^
5-Fu	80.54 ± 1.91^a^	4.12 ± 1.03^ab^	15.34 ± 1.79^a^
80 *µ*g mL^−1^	79.55 ± 1.09^ab^	5.56 ± 2.78^a^	14.89 ± 3.87^a^
160 *µ*g mL^−1^	80.95 ± 2.50^a^	6.00 ± 1.24^a^	13.05 ± 1.26^ab^

*Note.* HT-29 cells were treated with WPG for 48 h and analyzed by flow cytometry after staining with PI. The positive control was 5-Fu and negative control was purified water. Values with different letter superscripts in the same column are significantly different (*P* < 0.05).

**Table 3:** Primers used for apoptotic signaling genes.

Gene	Primers	
*β*-Actin	Forward	TCACCCTGAAGTACCCCATC
	Reverse	CCATCTCTTGCTGCAAGTCC
Bax	Forward	TCCACCAAGAAGCTGAGCGA
	Reverse	GTCCAGCCCATGATGGTTCT
Bad	Forward	CCTTTAAGAAGGGACTTCCTCGCC
	Reverse	ACTTCCGATGGGACCAAGCCTTCC
Bcl-xl	Forward	ATGGCAGCAGTAAAGCAAGCGC
	Reverse	TTCTCCTGGTGGCAATGGCG
Caspase-3	Forward	TTTGTTTGTGTGCTTCTGAGCC
	Reverse	ATTCTGTTGCCACCTTTCGG
Cyto-C	Forward	CCAGGACTGTATGTGGAGCG
	Reverse	CTTGAGGACCAGTGGGCTGT
